# Baseline data collections of lipopolysaccharide content in 414 herbal extracts and its role in innate immune activation

**DOI:** 10.1038/s41598-024-66081-2

**Published:** 2024-07-04

**Authors:** Vindy Tjendana Tjhin, Masataka Oda, Masashi Yamashita, Tomoko Iwaki, Yasuko Fujita, Koji Wakame, Hiroyuki Inagawa, Gen-Ichiro Soma

**Affiliations:** 1 Control of Innate Immunity, Collaborative Innovation Partnership, Takamatsu, 761-0301 Japan; 2 Department of Pharmacology, Faculty of Pharmaceutical Sciences, Hokkaido University of Science, Sapporo, 006-8585 Japan; 3 Research Institute for Healthy Living, Niigata University of Pharmacy and Applied Life Sciences, Niigata, 956-0841 Japan

**Keywords:** Lipopolysaccharide, Herbal extracts, Macrophage activation, Database, Immunochemistry, Innate immunity

## Abstract

Some herbal extracts contain relatively high amounts of lipopolysaccharide (LPS). Because orally administered LPS activates innate immunity without inducing inflammation, it plays a role as an active ingredient in herbal extracts. However, the LPS content in herbal extracts remains extensively unevaluated. This study aimed to create a database of LPS content in herbal extracts; therefore, the LPS content of 414 herbal extracts was measured and the macrophage activation potential was evaluated. The LPS content of these hot water extracts was determined using the kinetic–turbidimetric method. The LPS concentration ranged from a few ng/g to hundreds of μg/g (Standard *Escherichia coli* LPS equivalent). Twelve samples had a high-LPS-content of > 100 μg/g, including seven samples from roots and three samples from leaves of the herbal extracts. These samples showed high phagocytosis and NO production capacity, and further investigation using polymyxin B, an LPS inhibitor, significantly inhibited macrophage activation. This study suggests that some herbal extracts contain sufficient LPS concentration to activate innate immunity. Therefore, a new approach to evaluate the efficacy of herbal extracts based on their LPS content was proposed. A database listing the LPS content of different herbal extracts is essential for this approach.

## Introduction

Lipopolysaccharide (LPS) is a lipid and polysaccharide molecule found in the outer membrane of gram-negative bacteria^1,2^. LPS has long been considered an endotoxin owing to its wide use as a potent inflammation inducer because it binds to Toll-like receptor (TLR4)^3–6^ of immune cells and activates nuclear factor-kappa beta (NFκB)^7–9^ to cause inflammatory cytokines, including interleukin-1 beta (IL-1β)^10–12^, interleukin-6 (IL-6)^13^, and tumor necrosis factor alpha (TNFα)^14,15^, inducing severe fever, diarrhea, and shock when intravenously injected^16–20^. Furthermore, although oral administration of LPS does not induce inflammation in healthy subjects, it has been observed that disrupted barrier system and bacterial translation may occur in diseases with persistent inflammatory lesions in the intestinal tract and periodontal tissues. Experimental models in which persistent bacterial and LPS invasion in vivo induces systemic inflammation suggest the involvement of LPS in chronic inflammatory diseases, including lifestyle-related diseases^21^.

However, gram-negative bacteria with LPS are found in large amounts in the human intestinal tract^22^, skin^23,24^, and other organs in contact with the outside world without causing any inflammatory effects under healthy conditions^25^. The decreased number of these gram-negative bacteria in the intestinal tract resulting from the use of antibiotics causes a decrease in the amount of antimicrobial peptides 59^26,27^, making individuals more susceptible to infections^28,29^. Thus, LPS in the intestinal tract and skin has been suggested to play a beneficial role in maintaining health. Furthermore, the lack of exposure to LPS is associated with susceptibility to allergic and infectious diseases^30,31^. This shows that LPS have unknowingly been taken orally and transdermally to maintain our health.

In a previous study, it was revealed that LPS is present in many plants, including herbal extracts^32^. It also known that several LPSs are present in rice and wheat, which are staple foods, and that their ingestion confers functional properties. Additionally, *Pantoea agglomerans* was isolated as the dominant LPS symbiont in wheat^33^. Oral consumption of *Pantoea agglomerans* LPS (LPSp) enhanced phagocytosis of abdominal macrophages in mice, but this effect was not observed in TLR4-deficient mice^34^. This indicates that orally administered LPS promotes foreign body removal via innate immunity using TLR4. Furthermore, in disease prevention and treatment experiments, oral LPSp administration was found to enhance the effect of anticancer drugs^35^, promote the treatment of lung metastases^36^, inhibit itching in atopic dermatitis^25^, prevent atherosclerosis in apolipoprotein-E (ApoE)-deficient mice^37^, prevent dementia in brain diabetes-induced mice^38^ etc. Additionally, a recent study reported that orally administered LPS suppressed diabetic symptoms by increasing the expression of insulin signaling-related factors, especially adiponectin, in adipose tissue in type 2 diabetes mellitus, a disease supposedly LPS-induced^39^. Furthermore, LPSp has been confirmed to be highly safe in rats, with no adverse effects after oral administration at 2 g/kg body weight (BW) or higher^40^.

From the above-mentioned studies, LPS from ingested food is likely to activate and regulate innate immunity. Furthermore, considering its presence in herbal extracts, there is a possibility that the consumption of herbal extracts may activate the body innate immunity regulation. Herbal extracts are defined as naturally occurring unrefined substances from any part or parts of plants, animals, and other organisms with one or more active ingredients intended to alleviate, treat, or prevent diseases^41^. The above-mentioned wheat is a herbal extract listed in the “The Japanese standards for nonpharmacopoeial herbal extracts 2022” and is called Shobaku^42^. The overall health benefits of consuming herbal extracts are generally thought to be due to the low molecular weight of the active ingredient. However, a sufficient amount of LPS in the herbal extracts can activate the innate immune system; therefore, LPS should also be considered an active ingredient of herbal extracts. As the innate immune system-activating effect of orally administered LPS is coming to light^34^, LPS in herbal extracts as a component of the effects of Chinese herbal medicine deserves attention. Thus, a database of the LPS content in herbal extracts and food ingredients is required to make this concept common knowledge.

In 1992, our group screened approximately 60 plant samples, including herbal extracts, for their LPS content and found that some plants had a high LPS content of over 100 μg/g^32^. However, since then, little effort has been made to measure the LPS content in herbal extracts. Montenegro et al. was the first to report on LPS’s ability to activate macrophages, an innate immunity mechanism, in Kampo medicine^43^. In this study, they showed that the macrophage-activating component of Juzen-taiho-to, an immune-boosting Kampo medicine formulated from 10 herbal extracts, is correlated with the amount of LPS, which is obtained from symbiotic bacteria existing in one of its ingredients. Their study showed that LPS is a functional component that activates and controls macrophages (innate immunity) in Juzen-taiho-to; hence, LPS can be regarded as an active component of the innate immune system of numerous herbal extracts because most herbal extracts have symbiotic bacteria that supply LPS. Therefore, if information on the LPS content found in herbal extracts can be obtained, the knowledge that oral intake of LPS does not induce inflammation can be enforced, and a new perspective on the concept of LPS as an effective component of herbal extracts can be provided. However, data evaluating herbal extracts from the LPS viewpoint are currently extremely limited, as described above.

Thus, to provide a comprehensive list of the LPS content of herbal extracts and other food ingredients, the LPS content of 414 herbal extracts were measured and compared. Additionally, the macrophage activation potential of herbal extracts with particularly high-LPS-content was compared and measured to investigate the connection between LPS content and macrophage activity.

## Results

### Measurement of the LPS content of herbal extracts

**Figure 1:**
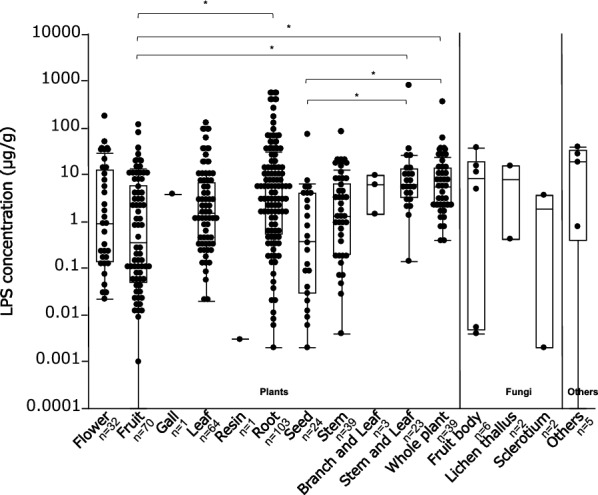
The distribution of the LPS concentration of the 414 samples measured using the Limulus reaction. The samples were divided into plants, fungi, and others. The plant samples were further categorized according to their parts. *p-value < 0.05 for Steel–Dwass test.

By measuring Limulus activity, the amount of LPS in the herbal extracts was examined. The LPS concentrations of 414 samples of herbal extracts obtained from vascular plants, fungi, and others ranging from below the detection limit to several 100 μg/g are shown in Table 1. Figure 1 shows the distributions of the LPS concentrations within each species. Herbal extracts from vascular plants were further divided according to their parts. For this analysis, the groups were classified according to the crude drug classification method. The results showed that herbal extract ingredients with high LPS contents were mostly found in the vascular plant group. Comparisons between vascular plant parts indicated that roots (107 samples) had significantly higher LPS levels than fruits (69 samples) and seeds (22 samples), and leaves (68 samples) had significantly higher LPS levels than fruits (69 samples). The average LPS concentration in all samples was 17.4 ± 69.3 μg/g. There are 12 samples containing high LPS concentration > 100 μg/g, 80 samples containing concentrations of 10–100 μg/g, and 162 samples containing concentrations of 1–10 ng/g. The 12 samples with significantly high LPS contents, which are listed in Table 2, were selected to further test the macrophage-activating effect of LPS. The measured LPS content indicated that herbal extracts contain LPS and that the amount of LPS in each plant’s part varies depending on the parts from which they are derived.

**Table 1:** LPS concentrations of 414 samples of herbal extracts. For herbal extracts with multiple scientific names, the scientific names listed in this table are those most used in Japan.

No	Species	English name	Scientific name	Part	Limulus activity (μg/g)
1	Plant	Achyranthes root	*Achyranthes bidentata* Blume	Root	0.391
2	Plant	Aconite root	*Aconitum carmichaelii* Debeaux	Root (Tuberous root)	2.705
3	Plant	Actinidia gall	*Actinidia polygama* (Siebold & Zucc.) Planch. ex Maxim	Fruit (Gall)	3.891
4	Plant	Adenophora root	*Adenophora triphylla* (Thunb.) A.DC	Root	5.984
5	Plant	Agarwood	*Aquilaria malaccensis* Lam	Stem (Xylem)	16.284
6	Plant	Agrimony	*Agrimonia eupatoria* L.	Stem and Leaf	3.282
7	Plant	Ajuga herb	*Ajuga decumbens *Thunb	Whole plant	10.642
8	Plant	Akebia fruit	*Akebia quinata* (Thunb. ex Houtt.) Decne	Fruit	12.147
9	Plant	Akebia stem	*Akebia quinata* (Thunb. ex Houtt.) Decne	Stem	1.962
10	Plant	Alfalfa	*Medicago sativa* L.	Stem and Leaf	4.089
11	Plant	Alisma tuber	*Alisma plantago-aquatica* subsp. *orientale* (Sam.) Sam	Root (Rhizome)	0.409
12	Plant	Allium chinense bulb	*Allium chinense* G.Don	Root (Bulb)	6.554
13	Plant	Allspice	*Pimenta dioica* (L.) Merr	Fruit	3.543
14	Plant	Amomum seed	*Wurfbainia villosa* var. *xanthioides* (Wall. ex Baker) Škorničk. & A.D.Poulsen	Seed	6.392
15	Plant	Amomum tsao-ko fruit	*Lanxangia tsao-ko* (Crevost & Lemarié) M.F.Newman & Škorničk	Fruit (Mature fruit)	0.018
16	Plant	Anemarrhena rhizome	*Anemarrhena asphodeloides* Bunge	Root (Rhizome)	38.905
17	Plant	Angelica	*Angelica archangelica* L.	Root	29.527
18	Plant	Angelica dahurica root	*Angelica dahurica* (Hoffm.) Benth. & Hook.f. ex Franch. & Sav	Root	266.554
19	Plant	Anise	*Pimpinella anisum* L.	Fruit	0.100
20	Plant	Apple	*Malus domestica* (Suckow) Borkh	Fruit	8.276
21	Plant	Apricot kernel	*Prunus armeniaca* L.	Seed	0.009
22	Plant	Aralia rhizome	*Aralia cordata* Thunb	Root (Rhizome)	503.986
23	Plant	Aralia root	*Aralia cordata* Thunb	Root	31.014
24	Plant	Aralia elata root bark	*Aralia elata* (Miq.) Seem	Root (Root bark)	3.891
25	Plant	Areca	*Areca catechu* L.	Seed	0.808
26	Plant	Arisaema tuber	*Arisaema heterophyllum* Blume	Root (Tuber)	1.488
27	Plant	Arnica flower	*Arnica montana* L.	Flower	29.527
28	Plant	Artemisia leaf	*Artemisia princeps* Pamp	Leaf	121.750
29	Plant	Artichoke	*Cynara cardunculus* L.	Stem and Leaf	4.642
30	Plant	Ash bark	*Fraxinus chinensis* subsp. *rhynchophylla* (Hance) A.E.Murray	Stem (Bark)	1.251
31	Plant	Ashitaba	*Angelica keiskei* (Miq.) Koidz	Leaf	0.220
32	Plant	Asparagus	*Asparagus officinalis* L	Stem	0.066
33	Plant	Asparagus root	*Asparagus cochinchinensis* (Lour.) Merr	Root	2.389
34	Plant	Aster root	*Aster tataricus* L.f.	Root (Root and Rhizome)	13.635
35	Plant	Astragalus root	*Astragalus mongholicus* Bunge	Root	1.256
36	Plant	Asunaro	*Thujopsis dolabrata* (L.f.) Siebold & Zucc	Branch and Leaf	1.445
37	Plant	Atractylodes lancea rhizome	*Atractylodes lancea* (Thunb.) DC	Root (Rhizome)	5.609
38	Plant	Bamboo culm	*Bambusa textilis* McClure	Stem (Culm)	1.151
39	Plant	Banaba	*Lagerstroemia speciosa* (L.) Pers	Leaf	0.363
40	Plant	Barbed skullcup herb	*Scutellaria barbata* D.Don	Whole plant	2.372
41	Plant	Barberry	*Berberis vulgaris* L.	Fruit	0.011
42	Plant	Bay leaf, Laurel	*Laurus nobilis* L.	Leaf	1.636
43	Plant	Bearberry leaf	*Arctostaphylos uva-ursi* (L.) Spreng	Leaf	0.735
44	Plant	Beautiful sweetgum fruit	*Liquidambar formosana* Hance	Fruit	0.778
45	Plant	Beet	*Beta vulgaris* L.	Root	0.124
46	Plant	Belvedere fruit	*Bassia scoparia* (L.) A.J.Scott	Fruit	7.322
47	Plant	Bilberry	*Vaccinium myrtillus* L.	Leaf	0.397
48	Plant	Birch, Abedul, Betula	*Betula pendula* Roth	Leaf	0.132
49	Plant	Bitter bottle gourd	*Cucurbita pepo* L.	Fruit	0.151
50	Plant	Bitter melon	*Momordica charantia* L.	Fruit	118.514
51	Plant	Bitter orange peel	*Citrus* × *aurantium* L.	Fruit (Peel)	0.050
52	Plant	Black tea	*Camellia sinensis* (L.) Kuntze	Leaf	1.075
53	Plant	Blackthorn	*Prunus spinosa* L.	Fruit	0.175
54	Plant	Bladder wrack	*Fucus evanescens* C.Agardh	Whole plant	4.945
55	Plant	Boldo, Boldus	*Peumus boldus* Molina	Leaf	0.156
56	Plant	Boston ivy, Japanese ivy	*Parthenocissus tricuspidata* (Siebold & Zucc.) Planch	Leaf	96.453
57	Plant	Brown rice	*Oryza sativa* L.	Seed	3.446
58	Plant	Bupleurum root	*Bupleurum falcatum* L.	Root	148.514
59	Plant	Burdock	*Arctium lappa* L.	Root	4.295
60	Plant	Burdock fruit	*Arctium lappa* L.	Fruit	8.577
61	Plant	Cabbage	*Brassica oleracea* L.	Leaf	0.257
62	Plant	Calendula, Marigold	*Calendula officinalis* L.	Flower	21.622
63	Plant	Calumba	*Jateorhiza palmata* (Lam.) Miers	Root	4.549
64	Plant	Caraway	*Carum carvi* L.	Fruit	2.004
65	Plant	Cardamon	*Elettaria cardamomum* (L.) Maton	Fruit	9.203
66	Plant	Carob, St. john’s bread	*Ceratonia siliqua* L.	Fruit (Bean pod)	0.009
67	Plant	Cassia seed	*Senna obtusifolia* (L.) H.S.Irwin & Barneby	Seed	0.020
68	Plant	Cassis, Black currant	*Ribes nigrum* L.	Fruit	0.033
69	Plant	Cassis, Black currant	*Ribes nigrum* L.	Leaf	26.649
70	Plant	Catalpa fruit	*Catalpa ovata* G.Don	Fruit	2.199
71	Plant	Catnip, Catmint	*Nepeta cataria* L.	Stem and Leaf	13.009
72	Plant	Cat’s whisker, Java tea	*Orthosiphon aristatus* (Blume) Miq	Leaf	11.472
73	Plant	Cauliflower	*Brassica oleracea* L.	Stem	0.128
74	Plant	Celandine	*Chelidonium majus* L.	Whole plant	32.824
75	Plant	Celery	*Apium graveolens* L.	Root	0.037
76	Plant	Celery seed	*Apium graveolens* L.	Seed	4.377
77	Plant	Chaenomeles fruit	*Pseudocydonia sinensis* (Dum.Cours.) C.K.Schneid	Fruit	3.088
78	Plant	Chamaecrista herb	*Chamaecrista nomame* (Makino) H.Ohashi	Whole plant	0.833
79	Plant	Chaste tree	*Vitex agnus-castus* L.	Fruit	4.124
80	Plant	Cherry bark	*Prunus jamasakura* (Makino) Siebold ex Koidz	Stem (Bark)	1.300
81	Plant	China berry	*Melia azedarach* L./*Melia azedarach* var. *subtripinnata* Miq	Leaf	0.331
82	Plant	Chinese blackberry, sweet tea	*Rubus chingii* var. *suavissimus* (S.K.Lee) L.T.Lu	Leaf	0.304
83	Plant	Chinese honeylocust spine	*Gleditsia sinensis* Lam.	Stem (Hook)	84.297
84	Plant	Chinese prickly ash	*Zanthoxylum simulans* Hance	Fruit (Peel)	13.399
85	Plant	Chinese pulsatilla root	*Pulsatilla chinensis* (Bunge) Regel	Root	58.676
86	Plant	Chokeberry	*Aronia melanocarpa* (Michx.) Elliott	Fruit	0.106
87	Plant	Chrysanthemum flower	*Chrysanthemum indicum* L.	Flower (Capitula)	13.399
88	Plant	Chundan, Kathala hibutu tea	*Salacia reticulata* Wight	Root (Root bark)	0.678
89	Plant	Cimicifuga rhizome	*Actaea dahurica* (Turcz. ex Fisch. & C.A.Mey.) Franch	Root (Rhizome)	13.349
90	Plant	Cimicifuga rhizome	*Actaea simplex* (DC.) Wormsk. ex Prantl	Root (Rhizome)	4.822
91	Plant	Cinnamon	*Neolitsea cassia* (L.) Kosterm	Stem (Bark)	0.047
92	Plant	Cinnamon bark (Crude drug)	*Neolitsea cassia* (L.) Kosterm	Stem (Bark)	12.386
93	Plant	Citrus peel	*Citrus* × *aurantium* L./*Citrus reticulata* Blanco	Fruit (Peel)	0.106
94	Plant	Citrus unshiu peel	*Citrus* × *aurantium* f. *deliciosa* (Ten.) M.Hiroe/*Citrus reticulata* Blanco	Fruit (Peel)	0.133
95	Plant	Clematis root	*Clematis terniflora* var. *mandshurica* (Rupr.) Ohwi	Root	2.953
96	Plant	Clove (Crude drug)	*Syzygium aromaticum* (L.) Merr. & L.M.Perry	Flower (Flower bud)	0.216
97	Plant	Clove	*Syzygium aromaticum* (L.) Merr. & L.M.Perry	Flower (Flower bud)	0.075
98	Plant	Club moss	*Lycopodium clavatum* L.	Whole plant	2.434
99	Plant	Cnidium monnieri fruit	*Cnidium monnieri* (L.) Cusson	Fruit	36.730
100	Plant	Cnidium rhizome	*Ligusticum officinale* (Makino) Kitag	Root (Rhizome)	5.264
101	Plant	Codonopsis root	*Codonopsis pilosula* (Franch.) Nannf	Root	0.322
102	Plant	Cola	*Cola nitida* (Vent.) Schott & Endl	Seed	0.121
103	Plant	Coltsfoot	*Tussilago farfara* L.	Leaf	2.162
104	Plant	Coltsfoot flower	*Tussilago farfara* L.	Flower (Flower bud)	1.916
105	Plant	Comfrey, Boneset	*Symphytum officinale* L.	Root	29.459
106	Plant	Comfrey, Boneset	*Symphytum officinale* L.	Whole plant	21.986
107	Plant	Common Curculigo rhizome	*Curculigo orchioides* Gaertn	Root (Rhizome)	0.778
108	Plant	Common ducksmeat herb	*Spirodela polyrhiza* (L.) Schleid	Whole plant	366.554
109	Plant	Common knotgrass herb	*Polygonum aviculare* L.	Whole plant	14.243
110	Plant	Common mullein, Great mullein	*Verbascum thapsus* L.	Stem and Leaf	2.649
111	Plant	Common reed	*Phragmites australis* (Cav.) Trin. ex Steud	Stem	6.554
112	Plant	Coptis rhizome	*Coptis japonica* (Thunb.) Makino	Root (Rhizome)	0.289
113	Plant	Coriander	*Coriandrum sativum* L.	Fruit	20.986
114	Plant	Corn silk	*Zea mays* L.	Flower (Flower’s style)	180.068
115	Plant	Cornflower	*Centaurea cyanus* L.	Flower	0.155
116	Plant	Cornus fruit	*Cornus officinalis* Siebold & Zucc	Fruit	0.043
117	Plant	Corydalis tuber	*Corydalis yanhusuo* (Y.H.Chou & Chun C.Hsu) W.T.Wang ex Z.Y.Su & C.Y.Wu	Root (Tuber)	8.796
118	Plant	Couch grass, Quack grass	*Elymus repens* (L.) Gould	Root (Rhizome)	392.635
119	Plant	Cowherb	*Gypsophila vaccaria* (L.) Sm	Seed	0.354
120	Plant	Crataegus fruit	*Crataegus cuneata* Siebold & Zucc	Fruit	0.244
121	Plant	Cumin	*Cuminum cyminum* L.	Fruit	80.054
122	Plant	Curcuma rhizome	*Curcuma zedoaria* (Christm.) Roscoe	Root (Rhizome)	65.608
123	Plant	Cyperus rhizome	*Cyperus rotundus* L.	Root (Rhizome)	2.801
124	Plant	Daisy fleabane	*Erigeron annuus* (L.) Pers	Whole plant	0.389
125	Plant	Damiana	*Turnera diffusa* Willd. ex Schult	Leaf	1.962
126	Plant	Dandelion	*Taraxacum* Weber	Root	4.945
127	Plant	Dayflower	*Commelina communis* L.	Whole plant	6.058
128	Plant	Devil’s claw	*Harpagophytum procumbens* (Burch.) DC. ex Meisn	Root (Tuber)	0.188
129	Plant	Dioscorea rhizome	*Dioscorea japonica* Thunb.	Root (Rhizome)	0.010
130	Plant	Dipsacus root	*Dipsacus asper* Wall. ex DC	Root	0.170
131	Plant	Echinacea	*Echinacea purpurea* (L.) Moench	Stem and Leaf	10.635
132	Plant	Elder	*Sambucus nigra* L.	Flower	9.359
133	Plant	English hawthorn	*Crataegus laevigata* (Poir.) DC	Leaf	36.730
134	Plant	Ephedra herb	*Ephedra sinica* Stapf	Stem	0.723
135	Plant	Epimedium herb	*Epimedium grandiflorum* var. *koreanum* (Nakai) K.Suzuki	Leaf	11.359
136	Plant	Erythrina bark	*Erythrina variegata* L.	Stem (Bark)	16.959
137	Plant	Eucalyptus	*Eucalyptus globulus* Labill.	Leaf	0.023
138	Plant	Eucommia bark	*Eucommia ulmoides* Oliv.	Stem (Bark)	0.188
139	Plant	Eucommia leaf	*Eucommia ulmoides* Oliv.	Leaf	2.791
140	Plant	Euodia fruit	*Tetradium ruticarpum* (A.Juss.) T.G.Hartley	Fruit	10.642
141	Plant	European verbena herb	*Verbena officinalis* L.	Stem	7.001
142	Plant	Eyebright	*Euphrasia officinalis* L.	Stem and Leaf	8.577
143	Plant	Feather cockscomb seed	*Celosia argentea* L.	Seed	2.791
144	Plant	Fennel	*Foeniculum vulgare* Mill.	Fruit	0.045
145	Plant	Fenugreek	*Trigonella foenum-graecum* L.	Stem and Leaf	36.351
146	Plant	Fermented black soybean	*Glycine max* (L.) Merr.	Seed	5.264
147	Plant	Feverfew	*Tanacetum parthenium* (L.) Sch.Bip.	Whole plant	8.714
148	Plant	Figwort flower Picrorhiza rhizome	*Neopicrorhiza scrophulariiflora* (Pennell) D.Y.Hong	Root (Rhizome)	1.678
149	Plant	Finger citron	*Citrus medica* L.	Fruit (Peel)	18.716
150	Plant	Flatstem milkvetch seed	*Phyllolobium chinense* Fisch.	Seed	4.295
151	Plant	Forsythia fruit	*Forsythia suspensa* (Thunb.) Vahl.	Fruit	10.530
152	Plant	Fortune windmill palm petiole	*Trachycarpus fortunei* (Hook.) H.Wendl.	Leaf	1.151
153	Plant	Fortune’s drynaria rhizome	*Drynaria roosii* Nakaike	Root (Rhizome)	423.041
154	Plant	Foxtail millet	*Setaria italica* (L.) P.Beauv.	Seed	0.006
155	Plant	Fragrant rosewood	*Dalbergia odorifera* T.C.Chen	Root (Heart wood)	1.418
156	Plant	Fragrant solomonseal rhizome	*Polygonatum odoratum* (Mill.) Druce	Root (Rhizome)	0.894
157	Plant	Frankincense	*Boswellia sacra* Flück.	Resin	0.003
158	Plant	French bean	*Phaseolus vulgaris* L.	Fruit (Bean pod)	0.936
159	Plant	Garden burnet root	*Sanguisorba officinalis* L.	Root (Root and Rhizome)	6.392
160	Plant	Gardenia fruit	*Gardenia jasminoides* J.Ellis	Fruit	0.014
161	Plant	Gastrodia tuber	*Gastrodia elata* Blume	Root (Tuber)	0.155
162	Plant	Gentiana macrophylla root	*Gentiana macrophylla* Pall.	Root	0.385
163	Plant	Geranium herb	*Geranium thunbergii* Siebold & Zucc. ex Lindl. & Paxton	Stem and Leaf	10.642
164	Plant	German chamomile	*Matricaria chamomilla* L.	Flower	0.322
165	Plant	Ginger	*Zingiber officinale* Roscoe	Root (Rhizome)	122.020
166	Plant	Ginkgo	*Ginkgo biloba* L	Leaf	6.936
167	Plant	Ginseng	*Panax ginseng* C.A.Mey	Root	0.023
168	Plant	Glechoma hederacea herb	*Glechoma grandis* (A.Gray) Kuprian.	Stem and Leaf	3.470
169	Plant	Glycyrrhiza	*Glycyrrhiza uralensis* Fisch. ex DC.	Root	3.101
170	Plant	Goldenrod	*Solidago virgaurea* subsp. *asiatica* (Nakai ex Hara) Kitam. ex Hara L.	Leaf	18.378
171	Plant	Gooseberry, European gooseberry	*Ribes uva-crispa* L.	Fruit	0.000
172	Plant	Gorgon euryale seed	*Euryale ferox* Salisb.	Seed	0.081
173	Plant	Grape	*Vitis* L.	Leaf	1.151
174	Plant	Green tea leaf	*Camellia sinensis* (L.) Kuntze.	Leaf	0.115
175	Plant	Guarana seed	*Paullinia cupana* Kunth.	Seed	5.058
176	Plant	Guava	*Psidium guajava* L.	Fruit	0.080
177	Plant	Gymnema	*Gymnema sylvestre* (Retz.) R.Br. ex Sm.	Leaf	0.346
178	Plant	Haichow Elsholtzia herb	*Elsholtzia splendens* var. *splendens*	Whole plant	3.839
179	Plant	Hairyveine agrimonia herb	*Agrimonia pilosa* Ledeb.	Whole plant	23.892
180	Plant	Heather	*Calluna vulgaris* (L.) Hull.	Flower (Flower bud)	4.124
181	Plant	Hedysarum root	*Hedysarum polybotrys* Hand.-Mazz.	Root	1.364
182	Plant	Henna	*Lawsonia inermis* L.	Leaf	1.104
183	Plant	Heterophylly false starwort root	*Pseudostellaria heterophylla* (Miq.) Pax.	Root (Tuberous root)	0.141
184	Plant	Hibiscus, Roselle	*Hibiscus sabdariffa* L.	Flower (Calyx)	0.030
185	Plant	Hollyhock	*Alcea rosea* L.	Flower	0.906
186	Plant	Hop strobile	*Humulus lupulus* L.	Flower	1.628
187	Plant	Horse chestnut	*Aesculus hippocastanum* L.	Leaf	0.397
188	Plant	Horseradish	*Armoracia rusticana* G.Gaertn., B.Mey. & Scherb.	Root	0.422
189	Plant	Horsetail, Field hare-tail	*Equisetum arvense* L.	Stem	0.639
190	Plant	Houttuynia herb	*Houttuynia cordata* Thunb.	Whole plant (Aerial part)	1.880
191	Plant	Hovenia seed or fruit	*Hovenia dulcis* Thunb.	Fruit	3.446
192	Plant	Hyssop	*Hyssopus officinalis* L.	Stem and Leaf	4.945
193	Plant	Immature citrus unshiu peel	*Citrus* × *aurantium* f. *deliciosa* (Ten.) M.Hiroe/*Citrus reticulata* Blanco	Fruit (Peel)	0.091
194	Plant	Immature orange fruit	*Citrus* × *aurantium* L.	Fruit	0.066
195	Plant	Indian madder root	*Rubia cordifolia* L.	Root	12.147
196	Plant	Indigo	*Isatis tinctoria* L.	Branch and Leaf	9.762
197	Plant	Inula flower	*Pentanema britannica* (L.) D.Gut.Larr., Santos-Vicente, Anderb., E.Rico & M.M.Mart.Ort	Flower (Capitula)	13.349
198	Plant	Ipe, Taheebo	*Handroanthus impetiginosus* (Mart. ex DC.) Mattos	Stem (Bark)	4.377
199	Plant	Ipecac	*Carapichea ipecacuanha* (Brot.) L.Andersson	Root	0.577
200	Plant	Isatis root	*Isatis tinctoria* L.	Root	0.373
201	Plant	Isodon herb	*Isodon japonicus* (Burm.f.) H.Hara	Stem and Leaf	5.270
202	Plant	Japanese angelica root	*Angelica acutiloba* (Siebold & Zucc.) Kitag.	Root	16.284
203	Plant	Japanese angelica tree	*Aralia elata* (Miq.) Seem	Stem	2.791
204	Plant	Japanese banana, Hardy banana	*Musa basjoo* Siebold & Zucc. ex Iinuma	Root	94.135
205	Plant	Japanese bush cherry	*Prunus japonica* Thunb.	Seed	1.300
206	Plant	Japanese gentian	*Gentiana scabra* Bunge.	Root (Root and Rhizome)	2.791
207	Plant	Japanese horse chestnut	*Aesculus turbinata* Blume.	Fruit	0.098
208	Plant	Japanese primrose	*Primula sieboldii* É.Morren	Flower	51.176
209	Plant	Japanese thistle root	*Cirsium japonicum* DC	Root	13.009
210	Plant	Japanese thyme	*Thymus quinquecostatus* Čelak	Leaf	11.359
211	Plant	Japanese torreya	*Torreya nucifera* (L.) Siebold & Zucc.	Seed	0.094
212	Plant	Japanese valerian	*Valeriana fauriei* Briq.	Root (Root and Rhizome)	9.203
213	Plant	Jasmine	*Jasminum* L.	Flower	0.044
214	Plant	Javanese turmeric	*Curcuma zanthorrhiza* Roxb.	Root (Rhizome)	13.399
215	Plant	Jujube	*Ziziphus jujuba* var. *inermis* (Bunge) Rehder	Fruit	0.123
216	Plant	Jujube seed	*Ziziphus jujuba* var. *spinosa* (Bunge) Hu ex H.F.Chow	Seed	0.002
217	Plant	Juniper berry	*Juniperus communis* L.	Fruit	0.019
218	Plant	Kava, Kava pepper	*Piper methysticum* G.Forst	Root	54.365
219	Plant	Kidachi aloe	*Aloe arborescens* Mill.	Leaf	1.174
220	Plant	Kombu	*Laminariaceae*	Root	1.117
221	Plant	Kuwagataso	*Veronica miqueliana* Nakai	Stem and Leaf	7.481
222	Plant	Lady’s mantle	*Alchemilla vulgaris* L.	Leaf	6.936
223	Plant	Lemon grass	*Cymbopogon citratus* (DC.) Stapf.	Stem and Leaf	0.142
224	Plant	Licorice	*Glycyrrhiza glabra* L.	Root	22.730
225	Plant	Ligusticum sinense rhizome	*Conioselinum anthriscoides* (H.Boissieu) Pimenov & Kljuykov	Root (Rhizome)	10.642
226	Plant	Ligustrum fruit	*Ligustrum lucidum* W.T.Aiton	Fruit	8.430
227	Plant	Lilium bulb	*Lilium lancifolium* Thunb.	Leaf (Bulb)	0.056
228	Plant	Linden, Lime flower	*Tilia cordata* Mill.	Leaf	3.891
229	Plant	Lindera root	*Lindera aggregata* var. *aggregata*	Root	61.986
230	Plant	Lithospermum root	*Lithospermum erythrorhizon* Siebold & Zucc.	Root	10.530
231	Plant	Long pepper	*Piper longum* L.	Fruit	1.063
232	Plant	Lonicera flower	*Lonicera japonica* Thunb.	Flower (Flower bud)	0.256
233	Plant	Lonicera leaf and stem	*Lonicera japonica* Thunb.	Stem and Leaf	2.162
234	Plant	Lophatherum herb	*Lophatherum gracile* Brongn.	Whole plant	1.628
235	Plant	Loquat leaf	*Eriobotrya japonica* (Thunb.) Lindl.	Leaf	0.084
236	Plant	Low-bush cranberry	*Vaccinium macrocarpon* Aiton	Leaf	3.268
237	Plant	Luffa, Vegetable sponge	*Luffa aegyptiaca* Mill.	Fruit (Fiber of mature fruit)	23.892
238	Plant	Luobuma	*Apocynum venetum* L.	Leaf	5.984
239	Plant	Lycium bark	*Lycium chinense *Mill.	Root (Root bark)	5.609
240	Plant	Lycium leaf	*Lycium chinense* Mill.	Branch and Leaf	6.155
241	Plant	Magnolia bark	*Magnolia obovata* Thunb.	Stem (Bark)	2.946
242	Plant	Magnolia flower	*Magnolia kobus* DC	Flower (Flower bud)	0.131
243	Plant	Mallotus bark	*Mallotus japonicus* (L.f.) Müll.Arg.	Stem (Bark)	3.088
244	Plant	Mallow	*Malva sylvestris* L.	Whole plant	5.469
245	Plant	Malt	*Hordeum vulgare* L.	Seed	73.500
246	Plant	Marjoram, Sweet marjoram	*Origanum majorana* L.	Leaf	0.141
247	Plant	Marshmallow, Altea	*Althaea officinalis* L.	Leaf	19.486
248	Plant	Marshmallow, Altea	*Althaea officinalis* L.	Root	8.276
249	Plant	Meadowsweet	*Filipendula ulmaria* (L.) Maxim	Stem and Leaf	26.486
250	Plant	Melia fruit	*Melia azedarach* L.	Fruit (Mature fruit)	0.049
251	Plant	Melilot	*Melilotus officinalis* (L.) Lam.	Whole plant	2.503
252	Plant	Mentha herb	*Mentha canadensis* L.	Leaf	6.199
253	Plant	Milk thistle	*Silybum marianum* (L.) Gaertn.	Whole plant	2.059
254	Plant	Mountain ash	*Sorbus aucuparia* L.	Fruit	0.121
255	Plant	Moutan bark	*Paeonia* × *suffruticosa* Andrews	Root (Root bark)	1.424
256	Plant	Mulberry	*Morus alba* L.	Leaf	3.101
257	Plant	Mulberry bark	*Morus alba* L.	Root (Root bark)	10.530
258	Plant	Mulberry fruit	*Morus alba* L.	Fruit	5.264
259	Plant	Myrobalan fruit	*Terminalia chebula* Retz.	Fruit	0.001
260	Plant	Nandina fruit	*Nandina domestica* Thunb.	Fruit	2.649
261	Plant	Natural indigo (Dye)	*Indigofera tinctoria* L.	Leaf	0.020
262	Plant	Nikko maple	*Acer maximowiczianum* Miq.	Stem (Bark)	0.906
263	Plant	Notopterygium	*Hansenia weberbaueriana* (Fedde ex H.Wolff) Pimenov & Kljuykov	Root (Rhizome)	2.946
264	Plant	Nutmeg	*Myristica fragrans* Houtt.	Seed	0.012
265	Plant	Oat	*Avena sativa* L.	Stem and Leaf	825.541
266	Plant	Olive	*Olea europaea* L.	Leaf	0.302
267	Plant	Ophiopogon root	*Ophiopogon japonicus *(Thunb.) Ker Gawl.	Root	0.220
268	Plant	Orange daylily	*Hemerocallis fulva* var. *fulva*	Flower (Flower bud)	0.022
269	Plant	Orange flower	*Citrus* × *aurantium* L.	Flower	0.919
270	Plant	Orange leaf	*Citrus* × *aurantium* L.	Leaf	30.878
271	Plant	Orange peel (bitter)	*Citrus* × *aurantium* L.	Fruit	0.015
272	Plant	Oregano	*Origanum vulgare* L.	Stem and Leaf	7.350
273	Plant	Oriental arborvitae leafy twig	*Platycladus orientalis *(L.) Franco.	Leaf	3.470
274	Plant	Orris root	*Iris florentina* L.	Root	12.327
275	Plant	Pale butterfly bush flower	*Buddleja officinalis* Maxim.	Flower (Flower bud)	0.208
276	Plant	Panax japonicus rhizome	*Panax japonicus* (T.Nees) C.A.Mey	Root (Rhizome)	0.322
277	Plant	Panax notoginseng root	*Panax notoginseng* (Burkill) F.H.Chen	Root	0.002
278	Plant	Parsley	*Petroselinum crispum* subsp. *crispum*	Leaf	0.627
279	Plant	Patrinia herb	*Patrinia scabiosifolia* Link	Whole plant	0.723
280	Plant	Peach	*Prunus persica *(L.) Batsch	Leaf	0.464
281	Plant	Pennyroyal	*Mentha pulegium* L.	Whole plant	0.389
282	Plant	Peony	*Paeonia lactiflora* Pall.	Flower	0.125
283	Plant	Peony root	*Paeonia lactiflora* Pall.	Root	0.529
284	Plant	Perilla fruit	*Perilla frutescens* var. *frutescens*	Fruit	0.084
285	Plant	Perilla herb	*Perilla frutescens* var. *crispa* (Thunb.) H.Deane	Leaf	4.650
286	Plant	Perilla, Beefsteak plant	*Perilla frutescens* (L.) Britton	Stem	5.264
287	Plant	Persimmon	*Diospyros kaki* L.f.	Leaf	1.474
288	Plant	Persimmon calyx	*Diospyros kaki* L.f.	Fruit (Calyx)	0.465
289	Plant	Peucedanum root	*Kitagawia praeruptora* (Dunn) Pimenov.	Root	1.628
290	Plant	Phellodendron bark	*Phellodendron amurense* Rupr.	Stem (Bark)	6.993
291	Plant	Pine	Pinus L.	Leaf	1.019
292	Plant	Pinellia tuber	*Pinellia ternata* (Thunb.) Makino	Root (Tuber)	1.872
293	Plant	Plantago herb	*Plantago asiatica* L.	Whole plant	2.642
294	Plant	Plantago seed	*Plantago asiatica* L.	Seed	0.385
295	Plant	Platycodon root	*Platycodon grandiflorus* (Jacq.) A.DC.	Root	2.199
296	Plant	Polygala root	*Polygala tenuifolia* Willd.	Root	0.075
297	Plant	Polygonatum rhizome	*Polygonatum falcatum* A.Gray	Root (Rhizome)	0.006
298	Plant	Polygonum root	*Reynoutria multiflora* (Thunb.) Moldenke	Root (Tuberous root)	0.008
299	Plant	Pomegranate rind	*Punica granatum* L.	Fruit (Peel)	0.777
300	Plant	Potentilla, Silverweed	*Argentina anserina* (L.) Rydb	Whole plant	6.731
301	Plant	Prickly pear cactus	*Opuntia* Mill.	Flower	1.174
302	Plant	Processed ginger	*Zingiber officinale* Roscoe	Root (Rhizome)	45.486
303	Plant	Processed mume	*Prunus mume* (Siebold) Siebold & Zucc.	Fruit	0.010
304	Plant	Prunella spike	*Prunella vulgaris* subsp. *asiatica* (Nakai) H.Hara	Flower (Spike)	35.216
305	Plant	Psoralea corylifolia fruit	*Cullen corylifolium* (L.) Medik.	Fruit	10.605
306	Plant	Pueraria Root	*Pueraria montana* var. *lobata* (Willd.) Maesen & S.M.Almeida ex Sanjappa & Predeep	Root	7.547
307	Plant	Purslane herb	*Portulaca oleracea* L.	Whole plant	13.055
308	Plant	Quercus bark	*Quercus acutissima* Carruth.	Stem (Bark)	6.836
309	Plant	Quercus salicina leaf	*Quercus salicina* Blume.	Leaf	0.259
310	Plant	Raspberry	*Rubus idaeus* L.	Leaf	9.614
311	Plant	Red clover	*Trifolium pratense* L.	Whole plant	7.507
312	Plant	Red poppy, Corn poppy	*Papaver rhoeas* L.	Flower	5.001
313	Plant	Rehmannia root	*Rehmannia glutinosa* (Gaertn.) DC	Root	0.529
314	Plant	Rhubarb	*Rheum palmatum* L.	Root (Rhizome)	1.364
315	Plant	Rice paper plant	*Tetrapanax papyrifer* (Hook.) K.Koch	Stem (Pith)	0.028
316	Plant	Rooibos	*Aspalathus linearis* (Burm.f.) R.Dahlgren	Leaf	94.135
317	Plant	Rose	*Rosa* L.	Flower (Flower bud)	0.028
318	Plant	Rose fruit	*Rosa multiflora* Thunb.	Fruit	2.196
319	Plant	Rosehips	*Rosa* L.	Fruit (Peel)	0.030
320	Plant	Rosemary	*Salvia rosmarinus *Spenn.	Leaf	1.555
321	Plant	Rosewood	*Dalbergia cochinchinensis* Pierre	Stem (Heart wood)	0.004
322	Plant	Rugosa rose flower	*Rosa rugosa* Thunb.	Flower (Flower bud)	0.156
323	Plant	Sacred lotus, Lotus	*Nelumbo nucifera* Gaertn.	Root (Rhizome node)	624.459
324	Plant	Safflower	*Carthamus tinctorius* L.	Flower	38.392
325	Plant	Salvia miltiorrhiza root	*Salvia miltiorrhiza* Bunge.	Root	10.530
326	Plant	Sambucus wood	*Sambucus williamsii* Hance.	Stem	2.515
327	Plant	Saposhnikovia root and rhizome	*Saposhnikovia divaricata *(Turcz. ex Ledeb.) Schischk	Root (Root and Rhizome)	4.650
328	Plant	Sappan wood	*Biancaea sappan* (L.) Tod.	Stem (Heart wood)	0.198
329	Plant	Sargentgloryvine stem	*Sargentodoxa cuneata* (Oliv.) Rehder & E.H.Wilson	Stem	1.488
330	Plant	Sarsaparilla	*Smilax purhampuy* Ruiz.	Root (Root and Rhizome)	24.176
331	Plant	Saussurea root	*Dolomiaea costus* (Falc.) Kasana & A.K.Pandey	Root	5.609
332	Plant	Schizonepeta spike	*Nepeta tenuifolia* Benth.	Flower (Spike)	0.596
333	Plant	Scisandra fruit	*Schisandra chinensis* (Turcz.) Baill	Fruit	0.022
334	Plant	Scrophularia root	*Scrophularia ningpoensis* Hemsl.	Root	1.916
335	Plant	Scutellaria root	*Scutellaria baicalensis* Georgi	Root	2.485
336	Plant	Sea buckthorn, Argasse	*Hippophae rhamnoides* L.	Fruit	0.024
337	Plant	Seaweed	*Sargassum fusiforme* (Harvey) Setchell	Whole plant	2.668
338	Plant	Senna Leaf	*Senna alexandrina* var. *alexandrina*	Leaf	0.206
339	Plant	Sesame	*Sesamum indicum* L.	Seed	2.004
340	Plant	Sheep sorrel	*Rumex acetosella* subsp. p*yrenaicus* (Pourr. ex Lapeyr.) Akeroyd	Whole plant	6.392
341	Plant	Shiny bugleweed	*Lycopus lucidus* Turcz. ex Benth.	Stem and Leaf	1.364
342	Plant	Siberian cocklebur fruit	*Xanthium strumarium* L.	Fruit	0.098
343	Plant	Siberian ginseng	*Eleutherococcus senticosus* (Rupr. & Maxim.) Maxim.	Root	35.216
344	Plant	Silktree Albizia bark	*Albizia julibrissin* Durazz.	Stem (Bark)	0.927
345	Plant	Sinomenium stem	*Sinomenium acutum* (Thunb.) Rehder et E.H.Wilson	Stem	9.065
346	Plant	Smilax rhizome	*Smilax glabra* Roxb.	Root (Rhizome)	0.047
347	Plant	Snowbell-leaf tickclover herb	*Grona styracifolia* (Osbeck) H.Ohashi & K.Ohashi	Stem and Leaf	13.399
348	Plant	Songaria cynomorium herb	*Cynomorium coccineum* subsp. *songaricum* (Rupr.) J.Léonard	Stem (Fleshy stem)	0.075
349	Plant	Sophora japonica flower	*Styphnolobium japonicum* (L.) Schott.	Flower (Flower bud)	7.350
350	Plant	Sophora root	*Sophora flavescens* Aiton	Root	4.822
351	Plant	Sophora subprostrata root	*Sophora tonkinensis* var. *tonkinensis*	Root	7.001
352	Plant	Sour cherry	*Prunus cerasus* L.	Fruit	1.608
353	Plant	Sparganium rhizome	*Sparganium stoloniferum* (Buch.-Ham. ex Graebn.) Buch.-Ham. ex Juz.	Root (Rhizome)	0.018
354	Plant	Spatholobus suberectus stem	*Spatholobus suberectus* Dunn.	Stem (Vine)	0.927
355	Plant	Spearmint	*Mentha spicata* L.	Whole plant (Aerial part)	2.389
356	Plant	Spicebush	*Lindera umbellata* Thunb.	Stem	0.168
357	Plant	Spreading Hedyotis herb	*Scleromitrion diffusum* (Willd.) R.J.Wang	Whole plant	36.730
358	Plant	Star anise	*Illicium verum* Hook.f.	Fruit	0.010
359	Plant	Stellaria herb	*Stellaria media* (L.) Vill. L.	Whole plant	22.730
360	Plant	Stevia	*Stevia rebaudiana* (Bertoni) Bertoni	Whole plant	1.488
361	Plant	Stinging nettle, Nettle	*Urtica dioica* L.	Leaf	61.986
362	Plant	Summer savory	*Satureja hortensis* L.	Leaf	2.649
363	Plant	Sunflower	*Helianthus annuus* L.	Flower	0.120
364	Plant	Sweet flag root	*Acorus calamus* L.	Root (Rhizome)	1.364
365	Plant	Sweet hydrangea leaf	*Hydrangea serrata* (Thunb.) Ser.	Leaf	0.853
366	Plant	Sweet tea vine, Gospel herb	*Gynostemma pentaphyllum* (Thunb.) Makino	Stem	0.399
367	Plant	Sweet violet	*Viola odorata* L.	Whole plant	61.986
368	Plant	Sweet woodruff	*Galium odoratum* (L.) Scop.	Leaf	9.203
369	Plant	Sweet wormwood herb	*Artemisia annua* L.	Whole plant (Aerial part)	1.138
370	Plant	Tansy	*Tanacetum vulgare* L.	Whole plant	14.878
371	Plant	Tarragon	*Artemisia dracunculus* L.	Leaf	2.860
372	Plant	Tetragonia herb	*Tetragonia tetragonoides* (Pall.) Kuntze.	Whole plant	18.716
373	Plant	Tokoro rhizome	*Dioscorea tokoro *Makino ex Miyabe	Root (Rhizome)	3.107
374	Plant	Tokyo violet herb	*Viola philippica* var. *philippica*	Whole plant	11.377
375	Plant	Tribulus fruit	*Tribulus terrestris* L.	Fruit	1.104
376	Plant	Trichosanthes fruit	*Trichosanthes kirilowii* Maxim.	Fruit	0.238
377	Plant	Trichosanthes peel	*Trichosanthes kirilowii* Maxim.	Fruit (Peel)	2.418
378	Plant	Trichosanthes root	*Trichosanthes kirilowii* Maxim.	Root	0.180
379	Plant	Trichosanthes seed	*Trichosanthes kirilowii* Maxim.	Seed	0.029
380	Plant	Trifoliate orange, Hardy orange	*Citrus trifoliata* L.	Fruit	0.058
381	Plant	Turmeric	*Curcuma longa* L.	Root (Rhizome)	21.095
382	Plant	Uncaria hook	*Uncaria rhynchophylla* (Miq.) Miq.	Stem (Hook)	3.891
383	Plant	Violet	*Viola* L.	Stem and Leaf	2.059
384	Plant	Walnut	*Juglans* L.	Fruit (Hull)	11.472
385	Plant	Walnut	*Juglans* L.	Leaf	6.561
386	Plant	Walnut	*Juglans regia* L.	Seed	0.033
387	Plant	Water chestnut	*Trapa natans* var. *bispinosa* (Roxb.) Makino	Fruit	2.953
388	Plant	Watercress	*Nasturtium officinale* R.Br.	Stem	20.041
389	Plant	Wheat	*Triticum aestivum* L.	Seed	0.244
390	Plant	White dead-nettle	*Lamium album* subsp. *barbatum* (Siebold & Zucc.) Mennema	Stem and Leaf	25.365
391	Plant	White horehound	*Marrubium vulgare* L.	Whole plant	7.782
392	Plant	White sandalwood	*Santalum album* L.	Stem (Xylem)	0.176
393	Plant	White willow	*Salix alba* L.	Stem (Bark)	0.373
394	Plant	Wild strawberry	*Fragaria vesca* L.	Leaf	2.286
395	Plant	Witch hazel, Hamamelis	*Hamamelis virginiana* L.	Leaf	1.256
396	Plant	Wormwood, Mugwort	*Artemisia princeps* Pamp.	Whole plant	2.059
397	Plant	Yarrow	*Achillea millefolium* L.	Whole plant	1.306
398	Plant	Yerbadetajo herb	*Eclipta prostrata* (L.) L.	Stem and Leaf	6.155
399	Plant	Yew	*Taxus brevifolia* Nutt.	Leaf	0.487
400	Fungus	Agaricus	*Agaricus blazei* Murill.	Fruit body	0.005
401	Fungus	Baikisei, Artist’s bracket	*Ganoderma applanatum* (Pers.) Pat.	Fruit body	12.312
402	Fungus	Ganoderma	*Ganoderma lucidum* P.Karsten	Fruit body	0.004
403	Fungus	Iceland moss	*Cetraria islandica* (L.) Ach.	Lichen thallus	15.527
404	Fungus	Jelly ear	*Auricularia auricula-judae* (Bull.) Quél	Fruit body	38.392
405	Fungus	Meshima	*Tropicoporus linteus* (Berk. & M.A.Curtis) L.W.Zhou & Y.C.Dai	Fruit body	4.945
406	Fungus	Polyporus sclerotium	*Polyporus umbellatus* Fries	Sclerotium	3.673
407	Fungus	Poria sclerotium	*Wolfiporia cocos* Ryvarden & Gilbertson (Poria cocos Wolf)	Sclerotium	0.002
408	Fungus	Snow tea	*Thamnolia vermicularis* (Sw.) Ach. ex Schaer	Lichen thallus	0.422
409	Fungus	Turkey tail	*Trametes versicolor* (L.) Lloyd.	Fruit body	11.472
410	Other (non-plant)	Abalone shell	*Haliotis diversicolor* Reeve, 1846	Shell	0.778
411	Other (non-plant)	Earthworm	*Pheretima aspergillum* Perrier	Whole body	18.716
412	Other (non-plant)	Spirulina	*Arthrospira platensis* Gomont.	Algae	39.662
413	Other (non-plant)	Trogopterus feces	*Trogopterus xanthipes* (Milne-Edwards)	Feces	28.014
414	Other (non-plant)	Water buffalo horn	*Bubalus bubalis*	Horn	0.000

**Table 2:** Twelve herbal extract samples with significantly higher LPS content than the other samples. The samples are listed in the order of high concentration.

Sample name	Scientific name	Part	Limulus activity (μg/g)
Oat	*Avena sativa* L.	Stem and leaf	825.5
Sacred lotus, Lotus	*Nelumbo nucifera* Gaertn.	Root	624.5
Aralia rhizome	*Aralia cordata* Thunb.	Root	504.0
Fortune’s drynaria rhizome	*Drynaria roosii* Nakaike	Root	423.0
Couch grass, Quack grass	*Elytrigia repens* (L.) Gould	Root	392.6
Common ducksmeat	*Spirodela polyrhiza* (L.) Schleid.	Leaf	366.6
Angelica dahurica root	*Angelica dahurica* (Hoffm.) Benth. & Hook.f. ex Franch. & Sav.	Root	266.6
Corn silk	*Zea mays* L.	Flower	180.1
Bupleurum root	*Bupleurum falcatum* L.	Root	148.5
Ginger	*Zingiber officinale* Roscoe.	Root	122
Artemisia leaf	*Artemisia princeps* Pamp.	Leaf	121.8
Bitter melon	*Momordica charantia* L.	Fruit	118.5

### Measurement of the macrophage activation potential of the herbal extracts

**Figure 2:**
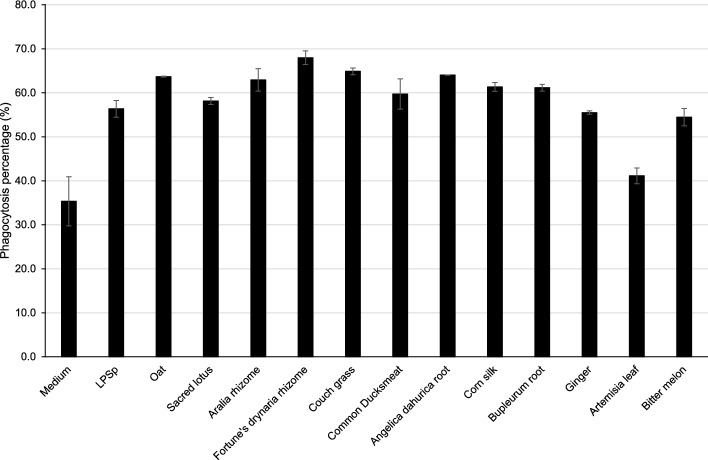
The percentage of phagocytic activity of RAW 264.7 cells stimulated by the 12 herbal extract samples containing the highest LPS levels are listed in Table 2. The concentrations of herbs and LPSp added were adjusted so that the LPS concentration was 100 ng/ml. The dotted line represents the phagocytosis percentage of RAW 264.7 cells without any external stimulation (medium only). Each bar represents the mean of two independent measurements, and the error bars represent the standard deviation.

Twelve herbal extract samples with LPS levels of ≥ 100 μg/g were tested for macrophage activation potential. Macrophage activation potential was assessed by measuring phagocytosis and nitric oxide (NO) production by stimulating RAW 264.7 cells with the herbal extracts. Stimulation using purified LPSp served as a positive control. Phagocytic activity was increased in all samples compared with that in the non-stimulated control group (Fig. 2). The phagocytosis ability of RAW 264.7 cells was increased when stimulated with Oat (*Avena sativa* L.), Sacred lotus (*Nelumbo nucifera* Gaertn.), Aralia rhizome (*Aralia cordata* Thunb.), Fortune’s drynaria rhizome (*Drynaria roosii* Nakaike), Couch grass (*Elytrigia repens* (L.) Gould), Angelica dahurica root (*Angelica dahurica*), Common ducksmeat (*Spirodela polyrhiza* (L.) Schleid.), Corn silk (*Zea mays* L.), and Bupleurum root (*Bupleurum falcatum* L.) compared with the positive control LPSp. The phagocytosis ability of RAW 264.7 cells with Ginger (*Zingiber officinale* Roscoe) was comparable, and that of Artemisia leaf (*Artemisia princeps* Pamp.) and Bitter melon (*Momordica charantia* L.) was lower than that of LPSp. The Pearson correlation between the amount of LPS and phagocytosis showed a clear positive correlation at R = 0.474. This suggests that LPS in crude drugs may increase the phagocytosis ability of macrophages, but other factors may also be involved.

**Figure 3:**
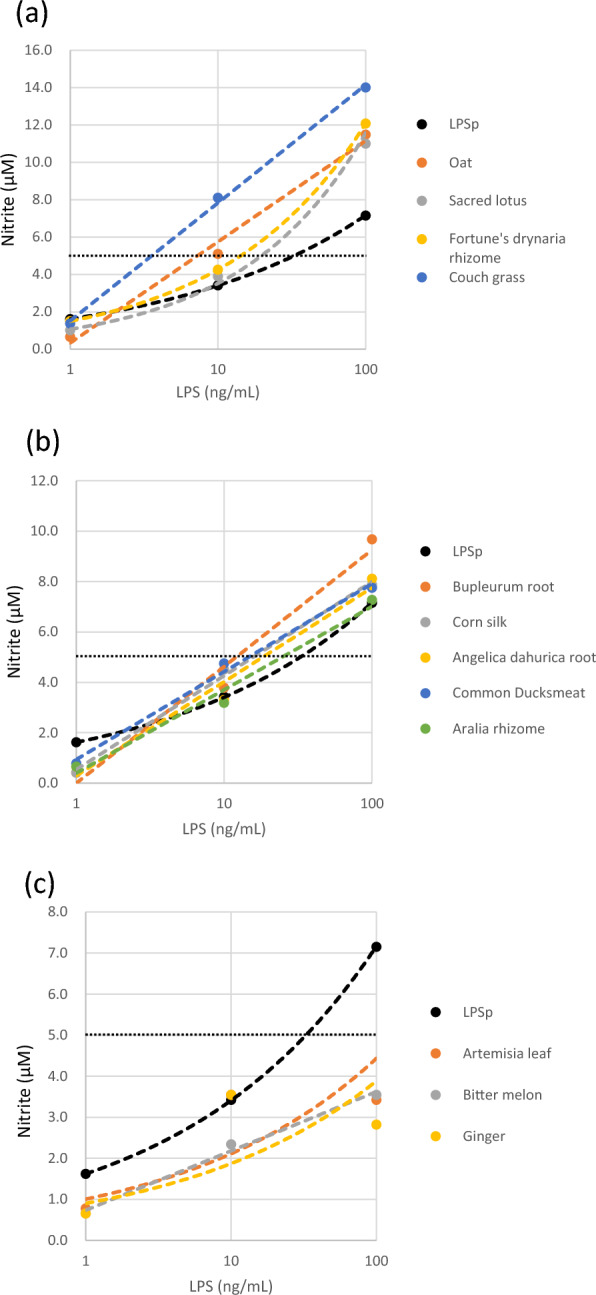
Dose–response curve of macrophage activation capacity determined by measuring the amount of NO produced as the amount of nitrite produced by RAW 264.7 cells stimulated by adding 1, 10, and 100 ng/ml per LPS to the 12 herbal extract samples containing the highest LPS content listed in Table 2. The amount of LPS needed to induce 5 µM more nitrite than LPSp used as control is (**a**) less than LPSp, (**b**) equivalent to LPSp, and (**c**) more than LPSp in this group. The dotted lines represent 5 µM Nitrite. The trendline equations (dashed lines) and R^2^ of each line are listed in Table 3.

To compare the NO production ability of the 12 herbal extracts with that of the positive control LPSp, the dose–response curves of the 12 herbal extract samples are presented in Fig. 3. The 12 herbal extracts were divided based on the amount of LPS required to induce 5 µM more nitrite than LPSp. Oat, (*Avena sativa* L.), Sacred lotus (*Nelumbo nucifera* Gaertn.), Fortune’s drynaria rhizome (*Drynaria roosii* Nakaike), and Couch grass (*Elytrigia repens* (L.) Gould) required a fewer samples per LPS content to induce 5 µM NO compared with LPSp (Fig. 3a). Corn silk (*Zea mays* L.), Bupleurum root (*Bupleurum falcatum* L.), Angelica dahurica root (*Angelica dahurica*), Common duckmeat (*Spirodela polyrhiza* (L.) Schleid.), and Angelica dahurica root (*Angelica dahurica*) required equivalent amounts of LPSp (Fig. 3b), whereas Ginger (*Zingiber officinale* Roscoe), Artemisia leaf (*Artemisia princeps* Pamp.), and Bitter melon (*Momordica charantia* L.) required more samples per LPS content to induce 5 µM Nitrite compared with LPSp (Fig. 3c). Table 3 shows the amount of LPS content in each herbal extract required to induce 5 µM NO and the relative NO induction strength compared with LPSp.

**Table 3:** The equivalent amount of herbal extracts per LPS content needed to induce 5 µM nitrite, which is the relative nitrite induction strength compared with LPSp. The trendline equations and R^2^ of each line in Fig. 3 are also listed.

Samples	LPS (ng/mL)/nitrite 5 µM	Relative NO induction strength (LPSp)	Trendline equation	R^2^ value
LPSp	32.8	1.0	y = 1.6218x^0.3226^	1.00
Oat	7.3	4.5	y = 2.3505ln(x) + 0.3357	0.99
Sacred lotus	19.7	1.7	y = 1.0709x^0.517^	0.99
Aralia rhizome	24.7	1.3	y = 1.4364ln(x) + 0.3958	0.98
Fortune’s drynaria rhizome	14.3	2.3	y = 1.4994x^0.4533^	1.00
Couch grass	3.6	9.2	y = 2.7422ln(x) + 1.5183	1.00
Common Ducksmeat	14.6	2.2	y = 1.5147ln(x) + 0.937	0.99
Angelica dahurica root	18.3	1.8	y = 1.6192ln(x) + 0.2956	0.97
Corn silk	15.8	2.1	y = 1.6192ln(x) + 0.5361	1.00
Bupleurum root	12.0	2.7	y = 2.011ln(x) − 0.0051	0.98
Ginger	222.7	0.1	y = 0.903x^0.3166^	0.64
Artemisia leaf	145.2	0.2	y = 1.006x^0.3221^	0.73

**Figure 4:**
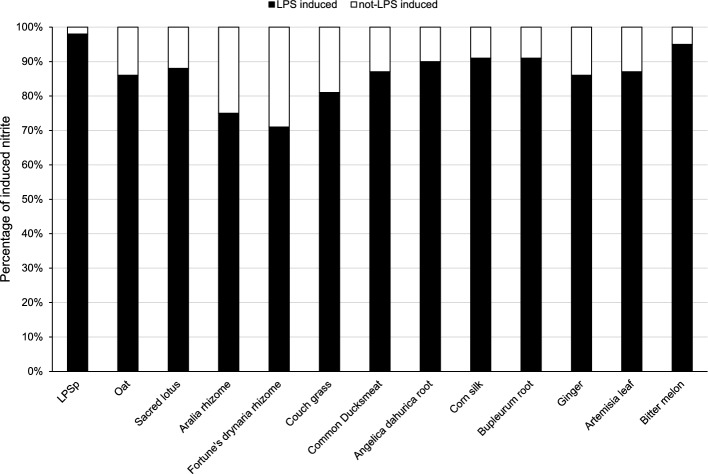
Macrophage activation potential determined by measuring the NO production of RAW 264.7 cells stimulated by the 12 herbal samples containing the highest LPS levels listed in Table 2. The percentage of NO produced by RAW 264.7 cells stimulated by LPS content (black bars) and other components (white) in the herbal extract samples. The concentrations of herbs and LPSp added were adjusted, making the LPS content 10 ng/ml. The black area represents the percentage of induced NO_2_ being decreased following polymyxin B addition, representing the percentage of NO_2_ induced by the LPS content in the herb samples. Each bar represents the mean of two independent measurements, and the error bars represent the standard deviation.

NO production results suggested that herbal extracts containing high LPS levels can activate macrophages. Moreover, NO production was significantly inhibited by the reaction with polymyxin B, an LPS inhibitor. In addition, an LPS inhibitor was used by Montenegro et al. as a way to verify that NO-inducing activity is obtained from LPS. The 12 samples exhibited significant inhibition of NO production, with inhibition rates of 71–95% (Fig. 4). The decrease in NO production when polymyxin was added suggests that it is mostly the LPS content that is involved in the macrophage-activating capacity of these herbal extracts.

## Discussion

Herbal extracts have several health-benefiting effects, such as hemostatic^44,45^, antifebrile^46,47^, detoxifying^48^, sweating^49^, and immunostimulating effects^50^, most of which are low molecular weight substances and have significantly contributed to the development of pharmaceuticals as the beginning of numerous medicines. LPS in herbal extracts supposedly causes this immunostimulating effect because previous LPS screening study revealed that some herbal extracts contain high LPS amounts (> 100 μg/g)^32^ and previous studies have shown that the oral intake of LPS enhances immunity and effectively prevents and improves various diseases, including cancer, viral infection, atopic dermatitis, diabetes, atherosclerosis, and Alzheimer’s disease^38,51–53^. Although there are more than several hundred herbal extracts worldwide and the possibility that the LPS in these herbal extracts playing a role in their functions is high, the LPS amount in them has never been measured or compared among the parts of plants from which they were obtained. Therefore, this study aimed to create a database of LPS levels in herbal extracts by measuring LPS levels in over 400 herbal extract samples stored at the Faculty of Pharmaceutical Sciences, Hokkaido University of Science, and to provide a basis for research to assess the immunostimulatory effects of herbal extracts and LPS’s contribution to these effects.

Table 1 shows the amount of LPS in 414 herbal extracts. LPS concentrations were widely distributed from a few μg/g to several hundred μg/g (Fig. 1). LPS content was shown to be significantly higher in roots (107 samples) than in fruits (69 samples) or seeds (22 samples) in terms of LPS concentration. Of the 414 herbal extracts measured in this study, approximately 100 herbal extracts contained ≥ 10 μg/g of LPS. Twelve of the herbal extracts exhibited very high LPS levels of over 100 μg/g. Comparison among vascular plant parts showed that the overall LPS level in root-derived herbal extracts was high and significantly higher than that in seed- and fruit-derived herbal extracts. Over half (seven) of the 12 high-LPS-content herbal extracts were root-derived. Most vascular plants are symbiotic with soil bacteria in their roots^54–56^. Symbiotic bacteria in soil promote plant growth through their involvement in nitrogen fixation, nutrient supply, and disease defense. Such bacteria are called plant growth-promoting rhizospheric microorganisms (PGPR)^57^; among them, bacteria of the genera *Pseudomonas*, *Azospirillum*, *Bradyrhizobium*, and *Rhizobium* are particularly essential. These bacteria are gram-negative bacteria and, therefore, may contribute to the high-LPS-content in the roots of herbal extracts. Montenegro et al. reported that 519 genera of bacteria are found in *Angelica sinensi*, a root-derived herbal extract that constitutes Juzen Daihoto, a Chinese herbal medicine known for its immunostimulating properties^43^. Among them, *Rahnella*, a gram-negative bacterium found in soil and fresh water, is abundant in *Angelica sinensi*. It was stipulated that the LPS content in *Angelica sinensi* is involved in the immunity-enhancing effects of Juzen Daihoto. The LPS content of *Angelica sinensi* (also called *Angelica acutiloba* Kitag. in Japan) was also measured in this study and it was shown that it contained 16 μg/g LPS, the 61st highest LPS content among all 414 samples in Table 1 (herb sample no. 202). These results suggest that the LPS amount in the root-derived herbal extract correlates with the number of soil-derived microorganisms that symbiotically coexist with the root-derived microorganisms during growth. These microorganisms are mostly gram-negative bacteria that contain a high LPS amount. On the other hand, the variation within each part group is large, suggesting that the high or low LPS content may not so much dependent on the part of the sample.

The amount of LPS contained in plants is considered to be derived from symbiotic bacteria. Therefore, the type and amount of symbiotic bacteria may vary depending on the origin of the plant, time of collection, variety, and cultivation method. Consequently, it is meaningful to measure multiple samples, but it is difficult to obtain multiple lots of crude drugs because most of them are imported. Therefore, we decided to use the variation in LPS content of one crude drug, brown rice, as a model for the variation in a single crude drug sample. In a previous study, we obtained brown rice from 15 different locations in Japan and measured LPS content in the 10.9 ± 4.3 μg/g range^58^. Although the LPS content of brown rice may not necessarily be universalizable to other crude drugs, we believe that this can be used as a reference value for the degree of variation in LPS content. The range of LPS content in this one sample was relatively stable compared to the range of 0.001–100 μg/g in the LPS content data (Table 1, Fig. 1) obtained for individual crude drugs. Therefore, based on this fact, we conducted the experiment with the belief that the approximate degree of LPS content could be evaluated with a single sample.

In this study, Limulus amebocyte lysate (LAL) test was used to detect LPS in the herbal extracts. However, it has been reported that β-1,3-glucan also reacts with LAL, so, there is a possibility of measuring plant-derived β-1,3-glucan contaminant with ordinary LAL. In this study, this contamination is prevented by using an LAL test kit containing a carboxymethylated curdlan which has reported act as a blocker of β-1,3-glucan mediated coagulation pathway^59^. Therefore, the limulus activity detected in this study were specific to LPS.

The macrophage-activating ability of LPS is a fundamental LPS action^34^. Therefore, the macrophage activation potential of herbal extracts by phagocytosis and NO production was assessed using macrophage-like RAW 264.7 cells. RAW246.7 cells transduce LPS signaling via TLR4^60^. In addition, many mammalian innate immune system cells, including humans, express TLR4^61^. Therefore, even though this study used mouse macrophage cells as a representative model, it is safe to assume that LPS contained in crude drugs is functional for mammals in general, including humans. However, further research is needed to determine the effects of LPS in humans, especially when administered orally. Twelve samples containing particularly high amounts of LPS (100 μg/g) were examined using these methods. The results showed that herbal extracts increased the phagocytosis capability of RAW 264.7 cells (Fig. 2). The NO production by RAW 264.7 cells caused by these samples was found to be higher, similar, or lower than purified LPSp, depending on the 12 herbal extracts (Fig. 3). The LPS itself in the group that exhibited higher activity may display high macrophage activation. However, it is speculated that a synergistic effect with macrophage activators, such as bacterial-derived nucleic acids, peptidoglycans, and flagellin, may be observed. Conversely, those that exhibited weaker activity than LPSp derived from *Enterobacteriaceae* may be because of the nature of the symbiotic gram-negative bacteria, as some LPSs, such as Bacteroides, are weak in biological activity, which depends on their lipid A structure^62,63^. Additionally, NO production was significantly (> 70%) reduced in all RAW 264.7 cells stimulated with 12 herbal extracts when polymyxin B, an LPS inhibitor, was added (Fig. 4). These results suggest that LPS is responsible for most of the macrophage activation potential of herbal extracts. However, the strength of the macrophage-activating ability of the herbal extracts is not proportional to the amount of LPS contained and may significantly differ among various symbiotic bacteria. Therefore, in studying the innate immune activation potential of herbal extracts, it is necessary to assess and clarify their unique qualities.

Herbal extracts are often prescribed in daily doses of 1–10 g^64,65^. Of the 414 herbal extracts for which LPS levels were measured in this study, 98 contained over 10 µg/g LPS, and oral intake of LPS increased the phagocytic activity of abdominal macrophages in mice at 10 µg/kg BW for 7 days^34^, induced increase in capillary vascularity at 10 µg/kg BW in human randomized control trial studies^66^, and in fish, 5–20 μg/kg BW increased the ability to prevent infection^67^. Based on these studies, 10 μg/kg BW of LPS can activate innate immunity, which is 500 μg/day for a 50 kg human. Therefore, consuming a daily dose of herbal extracts may mean taking in an effective amount of LPS, meaning that LPS may contribute to the medicinal effects of the herbal extracts. Juzen Daiho-to, a combination of herbal extracts, reportedly has preventive and ameliorative effects against diabetes and cancer partly because LPS is one of its ingredients^68,69^. The 414 herbal extract samples measured in this study are much greater than the 157 listed in the Japanese Pharmacopoeia. These should be sufficient populations for primary screening based on the efficacy of oral LPS intake over immune functions and the activation of immune cells using macrophages and other cells in herbal extracts. However, because the LPS content of plants is obtained from the symbiotic gram-negative bacterial population and may differ greatly depending on the time of collection, variety, cultivation method, etc., the LPS content of the samples to be studied should be analyzed with caution on a sample-by-sample basis.

## Methods

### Sample preparation

All dried samples were purchased from Tochimoto Tenkaido Co., Ltd. (Osaka, Japan). The dried samples were pulverized, and 100 mg powdered samples were extracted in 1 ml distilled water for 20 min at 90 °C. Subsequently, the samples were sonicated for 20 min and vortexed for two minutes to extract LPS. Next, the supernatants were obtained after centrifugation at 830 × *g* for 15 min. All methods involving the dried samples were carried out in accordance with relevant guidelines^70^.

### Measurement of the LPS contents of herbal extracts

The LPS concentration in the samples were assayed using the kinetic–turbidimetric method. All samples were diluted 10,000-fold using pyrogen-free distilled water. Sample supernatants (0.2 ml) were added to LAL-ES in a glass tube (Limulus ES-II single test; Wako Pure Chemical Industries Ltd., Osaka, Japan). After a few seconds of votexing, the gelation time was measured using a Toxinometer ET-6000 (Wako Pure Chemical Industries Ltd.), and the specific activity was calculated using an LS Toximaster (Wako Pure Chemical Industries Ltd.), a data acquisition program for the Toxinometer.

The LAL test kits of Wako contain carboxymethylated curdlan in freeze-dried reagents, which stops β-d-glucans from triggering an interference in the test. Therefore, this test kit used in this study is specific to LPS^59^.

### Phagocytosis assay

Phagocytic activity was measured using flow cytometry as previously described with minor modifications^71^. Briefly, the mouse macrophage/monocyte cell line RAW 264.7 cells (obtained from TIB-71; ATCC, Manassas, VA, USA) were treated for 18 h with extracts in a 48-well plate. The extract concentrations were measured so that the LPS content was 100 ng/ml. Next, fluorescent latex beads (Fluoresbrite^®^ YG Microspheres 1.0 μm; Polysciences, Warrington, PA) at a cell: bead ratio of 1:10 were added and incubated for one hour. Cells were washed to eliminate non-internalized particles and detached from the well plate with 0.25% trypsin treatment (Life Technologies, Carlsbad, CA, USA). The phagocytosis rate of the cells was measured using a Beckman Coulter Gallios flow cytometer and Kaluza software (Beckman Coulter, Indianapolis, IN).

### Nitric oxide (NO) production by murine macrophages

In a 48-well plate, cells from the mouse macrophage/monocyte cell line RAW 264.7 were plated at 8 × 105 cells/ml and treated with herbal extracts. The added extract concentrations were measured, so that the LPS content was 1, 10, and 100 ng/ml. The plate was incubated at 37 °C and 5% CO_2_. After 24-h incubation with extracts, the supernatants were collected, and the concentrations of nitrite (NO^2−^) released into the culture media were measured using Griess reagent. In addition, 100 μl Griess reagent was added to 100 μl diluted culture media in the wells of microtiter plates. After incubation at room temperature for ten minutes, absorbance at 570 nm was determined using an automated microplate reader (BIO-RAD, Hercules, CA, USA). The NO assay was conducted in duplicate. To determine the percentage of NO produced by the LPS in the herbal extracts, the concentrations of the extracts were measured, so that the LPS content was 10 ng/ml, and polymyxin B (Sigma-Aldrich, St. Louis, MO, USA) was added to each culture at a final concentration of 10 μg/ml.

### Statistical analysis

Data are presented as mean ± standard deviation (SD). Statistical analyses (Steel–Dwass test and Pearsons’ correlation) were performed using the JMP statistical software, version 17. 0. 0 (SAS Institute Inc., Cary, NC, USA). Statistical differences between multiple groups in the box-and-whisker plot were calculated using the Steel–Dwass test. A p-value < 0.05 was considered statistically significant. The line equation and its R^2^ value in Table 3 were performed using Microsoft Excel.

## Data Availability

All data generated or analyzed during this study are included in this published article.

## References

[1] Erridge C, Bennett-Guerrero E, Poxton IR (2002). Structure and function of lipopolysaccharides. Microbes Infect..

[2] Gorman A, Golovanov AP (2022). Lipopolysaccharide structure and the phenomenon of low endotoxin recovery. Eur. J. Pharm. Biopharm..

[3] Mazgaeen L, Gurung P (2020). Recent advances in lipopolysaccharide recognition systems. Int. J. Mol. Sci..

[4] Zamyatina A, Heine H (2020). Lipopolysaccharide recognition in the crossroads of TLR4 and caspase-4/11 mediated inflammatory pathways. Front. Immunol..

[5] Hoshino K (1999). Cutting edge: Toll-like receptor 4 (TLR4)-deficient mice are hyporesponsive to lipopolysaccharide: Evidence for TLR4 as the Lps gene product1. J. Immunol..

[6] Poltorak A (1998). Defective LPS signaling in C3H/HeJ and C57BL/10ScCr mice: Mutations in Tlr4 gene. Science.

[7] Garcia GE (2000). NF-κB-dependent fractalkine induction in rat aortic endothelial cells stimulated by IL-1β, TNF-α, and LPS. J. Leukoc. Biol..

[8] Vincenti MP, Burrell TA, Taffet SM (1992). Regulation of NF-κB activity in murine macrophages: Effect of bacterial lipopolysaccharide and phorbol ester. J. Cell. Physiol..

[9] Wijayanti N, Huber S, Samoylenko A, Kietzmann T, Immenschuh S (2004). Role of NF-kB and p38 MAP kinase signaling pathways in the lipopolysaccharide-dependent activation of heme oxygenase-1 gene expression. Antioxid. Redox Signal..

[10] Gattorno M (2007). Pattern of interleukin-1β secretion in response to lipopolysaccharide and ATP before and after interleukin-1 blockade in patients with CIAS1 mutations. Arthritis Rheum..

[11] Lopez-Castejon G, Brough D (2011). Understanding the mechanism of IL-1β secretion. Cytokine Growth Factor Rev..

[12] Lynch AM (2004). Lipopolysaccharide-induced increase in signalling in hippocampus is abrogated by IL-10—A role for IL-1β?. J. Neurochem..

[13] Bailly S, Ferrua B, Fay M, Gougerot-Pocidalo MA (1990). Differential regulation of IL 6, IL 1 A, IL 1β and TNFα production in LPS-stimulated human monocytes: Role of cyclic AMP. Cytokine.

[14] Agarwal S, Piesco NP, Johns LP, Riccelli AE (1995). Differential expression of IL-1β, TNF-α, IL-6, and IL-8 in human monocytes in response to lipopolysaccharides from different microbes. J. Dent. Res..

[15] Yoshimura A, Hara Y, Kaneko T, Kato I (1997). Secretion of IL-1β, TNF-α, IL-8 and IL-1ra by human polymorphonuclear leukocytes in response to lipopolysaccharides from periodontopathic bacteria. J. Periodontal Res..

[16] Miller SI, Ernst RK, Bader MW (2005). LPS, TLR4 and infectious disease diversity. Nat. Rev. Microbiol..

[17] Roth, J. & Blatteis, C. M. *Comprehensive Physiology* (ed. Terjung, R.) 1563–1604 (2014). 10.1002/cphy.c130033 25428854

[18] Wardill HR (2016). Irinotecan-induced gastrointestinal dysfunction and pain are mediated by common TLR4-dependent mechanisms. Mol. Cancer Ther..

[19] Zhan Z (2022). Overabundance of *Veillonella parvula* promotes intestinal inflammation by activating macrophages via LPS-TLR4 pathway. Cell Death Discov..

[20] Pålsson-McDermott EM, O'Neill LA (2004). Signal transduction by the lipopolysaccharide receptor, Toll-like receptor-4. Immunology.

[21] Festi D (2014). Gut microbiota and metabolic syndrome. World J. Gastroenterol..

[22] Hao, W.-L. & Lee, Y.-K. In *Public Health Microbiology: Methods and Protocols* (eds Spencer, J. F. T. & de Spencer, A. L. R.) 491–502 (Humana Press, 2004).

[23] Chiller K, Selkin BA, Murakawa GJ (2001). Skin microflora and bacterial infections of the skin. J. Investig. Dermatol. Symp. Proc..

[24] Percival SL, Emanuel C, Cutting KF, Williams DW (2012). Microbiology of the skin and the role of biofilms in infection. Int. Wound J..

[25] Nakai K, Kubota Y, Soma G-I, Kohchi C (2019). The effect of lipopolysaccharide-containing moisturizing cream on skin care in patients with mild atopic dermatitis. In Vivo.

[26] Jernberg C, Löfmark S, Edlund C, Jansson JK (2010). Long-term impacts of antibiotic exposure on the human intestinal microbiota. Microbiology.

[27] Nord CE, Edlund C (1990). Impact of antimicrobial agents on human intestinal microflora. J. Chemother..

[28] Brandl K (2008). Vancomycin-resistant enterococci exploit antibiotic-induced innate immune deficits. Nature.

[29] Lange K, Buerger M, Stallmach A, Bruns T (2016). Effects of antibiotics on gut microbiota. Dig. Dis..

[30] Braun-Fahrländer C (2002). Environmental exposure to endotoxin and its relation to asthma in school-age children. N. Engl. J. Med..

[31] Morcos M, Morcos W, Ibrahim M, Shaheen M (2011). Environmental exposure to endotoxin in rural and urban Egyptian school children and its relation to asthma and atopy. Minerva Pediatr..

[32] Inagawa H (1992). Homeostasis as regulated by activated macrophage. II. LPS of plant origin other than wheat flour and their concomitant bacteria. Chem. Pharm. Bull. (Tokyo).

[33] Dutkiewicz J, Mackiewicz B, Lemieszek MK, Golec M, Milanowski J (2016). *Pantoea agglomerans*: A mysterious bacterium of evil and good. Part IV. Beneficial effects. Ann. Agric. Environ. Med..

[34] Inagawa H (2016). Primed activation of macrophages by oral administration of lipopolysaccharide derived from *Pantoea agglomerans*. In Vivo.

[35] Hebishima T (2011). Oral administration of immunopotentiator from *Pantoea agglomerans* 1 (IP-PA1) improves the survival of B16 melanoma-inoculated model mice. Exp. Anim..

[36] Hirota K (2010). Antitumor effect of inhalatory lipopolysaccharide and synergetic effect in combination with cyclophosphamide. Anticancer Res..

[37] Kobayashi Y (2018). Oral administration of *Pantoea agglomerans*-derived lipopolysaccharide prevents development of atherosclerosis in high-fat diet-fed apoE-deficient mice *via* ameliorating hyperlipidemia, pro-inflammatory mediators and oxidative responses. PLoS One.

[38] Mizobuchi H (2021). Prevention of diabetes-associated cognitive dysfunction through oral administration of lipopolysaccharide derived from *Pantoea agglomerans*. Front. Immunol..

[39] Yamamoto K (2023). Oral administration of lipopolysaccharide enhances insulin signaling-related factors in the KK/Ay mouse model of type 2 diabetes mellitus. Int. J. Mol. Sci..

[40] Inagawa H, Kohchi C, Soma G-I (2011). Oral administration of lipopolysaccharides for the prevention of various diseases: Benefit and usefulness. Anticancer Res..

[41] Pal SK, Shukla Y (2003). Herbal medicine: Current status and the future. Asian Pac. J. Cancer Prev..

[42] *The Japanese Standards for Non-Pharmacopoeial Crude Drugs 2022* (National Institute of Health Sciences, 2022). 1364369

[43] Montenegro D (2015). Uncovering potential ‘herbal probiotics’ in Juzen-taiho-to through the study of associated bacterial populations. Bioorg. Med. Chem. Lett..

[44] Ebrahimi F, Torbati M, Mahmoudi J, Valizadeh H (2020). Medicinal plants as potential hemostatic agents. J. Pharm. Pharm. Sci..

[45] Ohkura N, Yokouchi H, Mimura M, Nakamura R, Atsumi G (2015). Screening for hemostatic activities of popular Chinese medicinal herbs in vitro. J. Intercult. Ethnopharmacol..

[46] Dogra S, Singh J, Koul B, Yadav D (2023). Artemisia vestita: A folk medicine with hidden herbal fortune. Molecules.

[47] Zhang H, Hai GF, Zhang C (2011). Experimental studies on analgesia and anti-febrile effects of the different extracts from radix *Angelicae dahuricae*. J. Xinxiang Med. Coll..

[48] Peter K (2013). A novel concept for detoxification: Complexation between aconitine and liquiritin in a Chinese herbal formula (‘Sini Tang’). J. Ethnopharmacol..

[49] Chiu S-C (2009). The therapeutic effect of modified Yu Ping Feng San on idiopathic sweating in end-stage cancer patients during hospice care. Phytother. Res..

[50] Lee AN, Werth VP (2004). Activation of autoimmunity following use of immunostimulatory herbal supplements. Arch. Dermatol..

[51] Kobayashi Y (2018). Oral administration of *Pantoea agglomerans*-derived lipopolysaccharide prevents metabolic dysfunction and Alzheimer’s disease-related memory loss in senescence-accelerated prone 8 (SAMP8) mice fed a high-fat diet. PLoS One.

[52] Wakame K, Komatsu K, Inagawa H, Nishizawa T (2015). Immunopotentiator from *Pantoea agglomerans* prevents atopic dermatitis induced by dermatophagoides farinae extract in NC/Nga mouse. Anticancer Res..

[53] Fukasaka M (2015). A lipopolysaccharide from pantoea agglomerans is a promising adjuvant for sublingual vaccines to induce systemic and mucosal immune responses in mice via TLR4 pathway. PLoS One.

[54] Bottini R, Cassán F, Piccoli P (2004). Gibberellin production by bacteria and its involvement in plant growth promotion and yield increase. Appl. Microbiol. Biotechnol..

[55] Luca B, Richard DB, Edward ADM, Alexandre B (2014). Linking soil microbial communities to vascular plant abundance along a climate gradient. New Phytol..

[56] Van Der Heijden MGA (2006). Symbiotic bacteria as a determinant of plant community structure and plant productivity in dune grassland. FEMS Microbiol. Ecol..

[57] Vejan P, Abdullah R, Khadiran T, Ismail S, Nasrulhaq Boyce A (2016). Role of plant growth promoting rhizobacteria in agricultural sustainability—A review. Molecules.

[58] Inagawa H (2016). Dewaxed brown rice contains a significant amount of lipopolysaccharide pointing to macrophage activation *via* TLRs. Anticancer Res..

[59] Tsuchiya M, Takaoka A, Tokioka N, Matsuura S (1990). Development of an endotoxin-specific Limulus amebocyte lysate test blocking β-glucan-mediated pathway by carboxymethylated curdlan and its application. Nippon Saikingaku Zasshi.

[60] Pi J (2014). Detection of lipopolysaccharide induced inflammatory responses in RAW264.7 macrophages using atomic force microscope. Micron.

[61] Vaure C, Liu Y (2014). A comparative review of toll-like receptor 4 expression and functionality in different animal species. Front. Immunol..

[62] Alexander C, Zahringer U, Kokubo S, Suda Y (2002). Chemical structure of lipid A-the primary immunomodulatory center of bacterial lipopolysaccharides. Trends Glycosci. Glycotechnol..

[63] Vatanen T (2016). Variation in microbiome LPS immunogenicity contributes to autoimmunity in humans. Cell.

[64] Jurenka JS (2009). Anti-inflammatory properties of curcumin, a major constituent of *Curcuma longa*: A review of preclinical and clinical research. Altern. Med. Rev..

[65] Schmeda-Hirschmann G, Yesilada E (2005). Traditional medicine and gastroprotective crude drugs. J. Ethnopharmacol..

[66] Nakata Y (2018). Effects of 3 months continuous intake of supplement containing *Pantoea agglomerans* LPS to maintain normal bloodstream in adults: Parallel double-blind randomized controlled study. Food Sci. Nutr..

[67] Kadowaki T (2013). Orally administered LPS enhances head kidney macrophage activation with down-regulation of IL-6 in common carp (*Cyprinus carpio*). Fish Shellfish Immunol..

[68] Ishikawa S (2012). Suppressive effect of juzentaihoto on vascularization induced by B16 melanoma cells in vitro and in vivo. Evid. Based Complement. Altern. Med..

[69] Ishida T (2022). Juzentaihoto suppresses muscle atrophy in KKAy mice. Biol. Pharm. Bull..

[70] Inagawa H (1992). Homeostasis as regulated by activated macrophage. II. LPS of plant origin other than wheat flour and their concomitant bacteria. Chem. Pharm. Bull..

[71] Yamamoto K (2020). Attempt to construct an in vitro model of enhancement of macrophage phagocytosis via continuous administration of LPS. Anticancer Res..

